# Vitamin E is necessary for zebrafish nervous system development

**DOI:** 10.1038/s41598-020-71760-x

**Published:** 2020-09-21

**Authors:** Brian Head, Jane La Du, Robyn L. Tanguay, Chrissa Kioussi, Maret G. Traber

**Affiliations:** 1 Linus Pauling Institute, Oregon State University, 307 LPSC, Corvallis, OR USA; 2 Molecular and Cell Biology Program, Oregon State University, Corvallis, OR USA; 3 Department of Environmental Toxicology, College of Agricultural Sciences, Oregon State University, Corvallis, OR USA; 4 Department of Pharmaceutical Sciences, College of Pharmacy, Oregon State University, Corvallis, OR USA; 5 School of Biological and Population Health Sciences, College of Public Health, Oregon State University, Corvallis, OR USA

**Keywords:** Biochemistry, Developmental biology, Neurogenesis

## Abstract

Vitamin E (VitE) deficiency results in embryonic lethality. Knockdown of the gene *ttpa* encoding for the VitE regulatory protein [α-tocopherol transfer protein (α-TTP)] in zebrafish embryos causes death within 24 h post-fertilization (hpf). To test the hypothesis that VitE, not just α-TTP, is necessary for nervous system development, adult 5D strain zebrafish, fed either VitE sufficient (E+) or deficient (E−) diets, were spawned to obtain E+ and E− embryos, which were subjected to RNA in situ hybridization and RT-qPCR. *Ttpa* was expressed ubiquitously in embryos up to 12 hpf. Early gastrulation (6 hpf) assessed by *goosecoid* expression was unaffected by VitE status. By 24 hpf, embryos expressed *ttpa* in brain ventricle borders, which showed abnormal closure in E− embryos. They also displayed disrupted patterns of *paired box 2a* (*pax2a*) and *SRY-box transcription factor 10* (*sox10*) expression in the midbrain-hindbrain boundary, spinal cord and dorsal root ganglia. In E− embryos, the collagen sheath notochord markers (*col2a1a* and *col9a2*) appeared bent. Severe developmental errors in E− embryos were characterized by improper nervous system patterning of the usually carefully programmed transcriptional signals. Histological analysis also showed developmental defects in the formation of the fore-, mid- and hindbrain and somites of E− embryos at 24 hpf. *Ttpa* expression profile was not altered by the VitE status demonstrating that VitE itself, and not *ttpa*, is required for development of the brain and peripheral nervous system in this vertebrate embryo model.

## Introduction

Vitamin E (VitE) is necessary during embryo development and prevents fetal resorption in VitE-deficient rats^1^. We have previously shown that VitE deficiency dysregulates whole animal phospholipid metabolism, energy status and antioxidant systems using VitE deficient zebrafish (*Danio rerio*) embryos^2,3^. VitE deficient vertebrates exhibit neurodevelopmental defects^4^, including exencephaly^5^, increased dorsal root ganglia (DRG) turnover^6^ and defective blood brain barriers^7^. By contrast, VitE supplementation confers protective effects against oxidative stress-induced neural tube defects (NTD) in mice^8^ and in human diabetic embryopathy^9^.

NTDs are a broad class of embryologic defects that occur during development; human embryos experience NTD onset between 22 and 30 days post-fertilization (dpf) ^10^, rats between 9 and 12 dpf^11^, and at the embryological period similar to zebrafish embryos less than 24 h post-fertilization (hpf) ^12^. By this developmental stage, the gene *ttpa* encoding for the α-tocopherol transfer protein (α-TTP) is expressed in the zebrafish embryo brain, eye and tailbud^13^, which suggests that these developing tissues require VitE and/or α-TTP. We have previously shown that a morpholino *ttpa* knockout at the one cell stage impairs brain and eye formation in zebrafish embryos^13^. Thus, either α-TTP, its ligand VitE or both are required for proper nervous system development.

The accepted α-TTP function is that it is involved in hepatic VitE trafficking, where it facilitates α-tocopherol secretion into the plasma^14^. In humans, *Ttpa* genetic defects cause ataxia with VitE deficiency (AVED). This disorder results in VitE-deficiency in early childhood and a sensory neuropathy caused by dying degenerative peripheral nerves^15,16^. Studies in mice have shown that VitE deficiency causes a localized degeneration of cerebellar Purkinje neurons and Bergmann glial cells^17^ and spinal cord neurons^18^. VitE supplementation in humans can prevent or halt progression of the disorder^19^. Thus, there is a strong connection between nervous system health and VitE sufficiency, nonetheless it is not clear how VitE may be critical during embryonic neurodevelopment.

We posit that there are several steps where VitE may be essential during neurodevelopment. Early brain development is a coordinated process that begins as early as gastrulation and is regulated by a complex set of cell signaling pathways that stimulate proliferation, differentiation and specification of neuronal cells and eventually tissue formation. Of note, neurulation differs between vertebrates; primary neurulation whereby the neural plate folds forming a tube in mammals contrasts with zebrafish neural keel formation which consists of a thickening of the neuroectoderm into a rod that subsequently cavitates forming the neural tube^20^. Secondary neurulation is similar between vertebrates and occurs in the caudal embryo by inflation of neural rod, epithelial transition to form a lumen and an expanded neural tube. Regardless, cell types and tissue layers are similarly derived with conserved cellular and molecular developmental mechanisms^21^.

Specifically in zebrafish, the neural progenitor cell populations are induced during gastrulation, as the embryonic shield forms and the neuroectoderm involutes^22,23^. Early neuroectoderm in zebrafish embryos is marked by the expression of goosecoid (*gsc*)^24^. Following the formation of the neural plate, neural crest (NC) cells migrate and give rise to neurons of the peripheral and enteric nervous system, craniofacial cartilage and bone, and even pigment cells^25–27^. Neurodevelopmental progression is well-studied with numerous pathways and genes identified as critical to development of the neural keel, neural tube, brain regionalization and more. For example, paired box transcription factor 2a (*pax2a*) is expressed in the midbrain-hindbrain boundary (mhb), neural keel, optic vesicle and pronephric mesoderm when the animal has developed 1–4 somites^28,29^. These *pax2a* expressing structures continue to form the isthmic organizer, neural tube, otic placode and pronephric ducts up to 24 hpf^30–32^. SRY-box transcription factor 10 (*sox10)* expression begins during gastrulation and is primarily localized to cranial and migratory NC between 12 and 24 hpf^25,33^. *Sox10* is a member of the SoxE family of genes, a group of transcription factors required for NC survival and migration^34^. In addition to NC, SoxE genes are responsible for regulation of collagen involved in cartilage formation, including the critical notochord-specific gene, *col2a1a*^35^*.* NC migration and differentiation is highly dependent on nutrient status^36,37^. Both *pax2a* and *sox10* expression patterns are irregular in oxidative stress models^38–40^ and errors in neurogenesis are marked by transcriptional changes of the Pax^41–43^ and SoxE family of genes^43–46^.

We hypothesized that VitE deficient zebrafish would experience nervous tissue disruption during embryogenesis. Prior studies from our lab have shown that impairing *ttpa* translation causes neural and eye tissue deformations, and by 24 hpf results in 100% zebrafish embryo death^13^. These data suggest that either α-TTP itself is necessary, or that its functional role to deliver VitE is essential for neurogenesis. Thus, we have sought to identify VitE-dependent processes by localizing gene expression of key neurogenesis markers in VitE deficient (E−) and sufficient (E+) embryos. To establish the role of VitE in support of neurodevelopment at two specific developmental time points, the relative gene expressions of specific markers in E+ and E− embryos were quantified at 12- and 24 hpf. Additionally, a comparative histological analysis was performed to assess E+ and E− embryo developmental defects at 24 hpf.

## Results

### Morphological abnormalities associated with VitE deficiency

**Figure 1:**
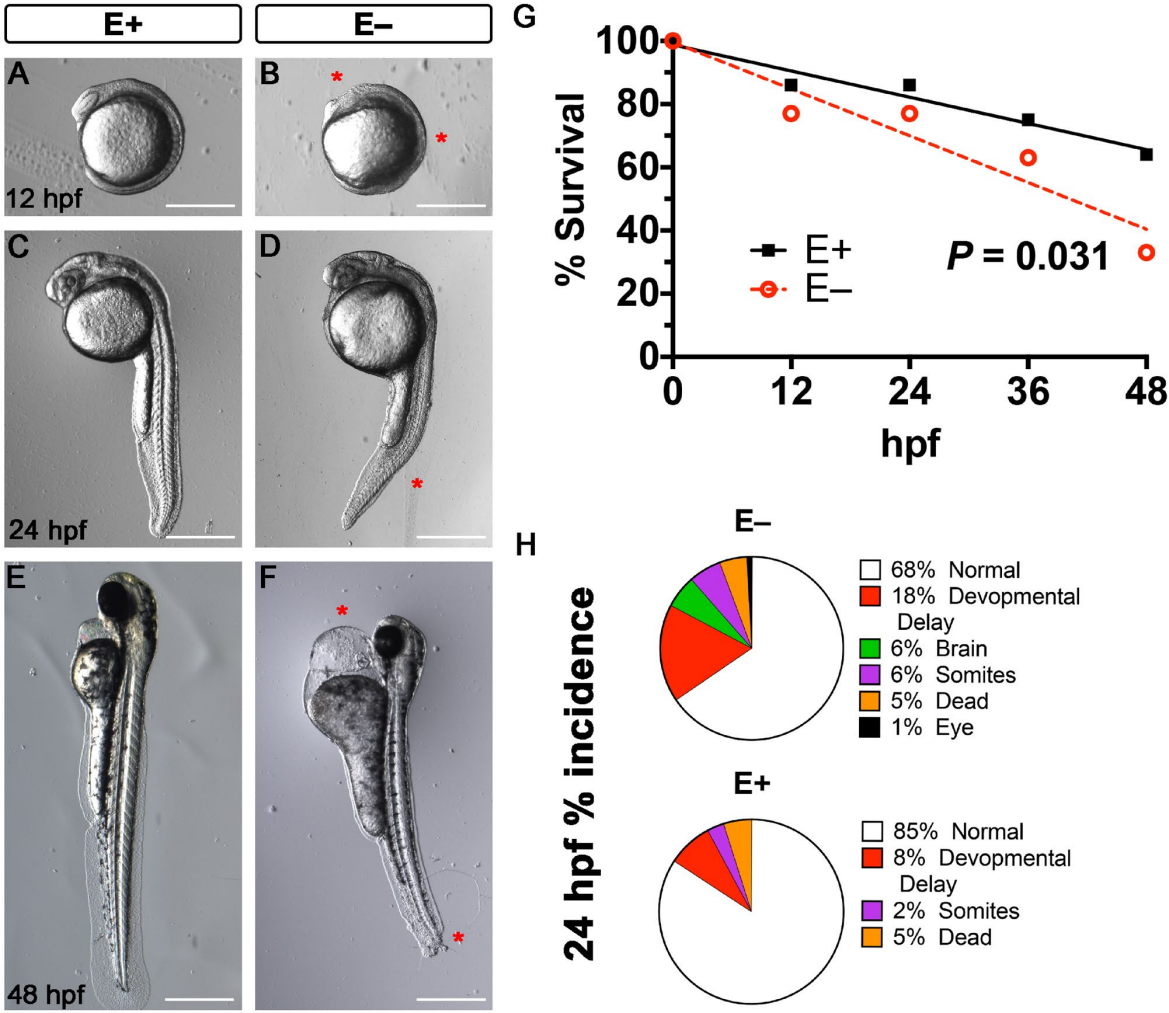
Morphological abnormalities associated with VitE deficiency at 12-, 24- and 48 h post-fertilization. Representative bright field images of E+ and E− embryos showed normal development in E+ embryos and abnormalities in the E− embryos. At 12 hpf, E+ embryos (**A**) had defined somites and eyes, while E− embryos (**B**) showed abnormal somite formation and a reduced dorsal region where the eye is usually located. At 24 hpf, E+ embryos (**C**) had clearly defined eyes, heads and somites, while the E− embryos (**D**) had pericardial edema, less well-defined somites and notochord. At 48 hpf, E+ embryos (**E**) had extended fins and pigmentation throughout the body, while the E− embryos (**F**) experienced severe pericardial edema, stunted fin formation; some E− embryos experience errors in tail formation. (**G**) Early mortality, defined as nonviable beyond that time point, was increased in E− embryos at 12 hpf. By 48 hpf only about 35% of the original E− clutch survived with about 75% of E+ embryos surviving (*P* < 0.031) At 24 hpf (**H**), E− embryos that were alive experienced greater incidences of developmental delay, as well as morphological malformations that include brain, eye, and somites deformities. *Indicate delay or defects in E− embryos relative to E+ . Scale bar represents 500 m. Representative embryos are shown. Figure panels (**A**–**F**) were generated with the BZ- × 700 microscope, processed with BZ-X Analyzer Software with image adjustments made with Adobe Photoshop v21.2.1.

The E− embryos experienced severe developmental morbidity and mortality outcomes, including deformation of the brain and eye and ill-defined somites by 12 hpf (Fig. 1A,B). By 24 hpf, surviving E− embryos displayed ill-defined somites and stunted fin formation (Fig. 1C,D). By 48 hpf, surviving E− embryos experienced severe pericardial and yolk sac edema, as well as disruption to tail development (Fig. 1E,F). Overall, mortality was greater (*P* = 0.031) in the E− relative to E+ embryos over the first 48 hpf (Fig. 1G). Similar to our previous report^3^, E− compared with E+ embryos experienced a greater incidence of severe brain and somite malformations and increased developmental delay (Fig. 1H). Malformations of the brain and eye were unique to E− embryos at 24 hpf. Notably, the E+ mortality was not significantly different from the mortality rate of embryos from parents fed a standard lab diet^47^.

### *Ttpa* localization

**Figure 2:**
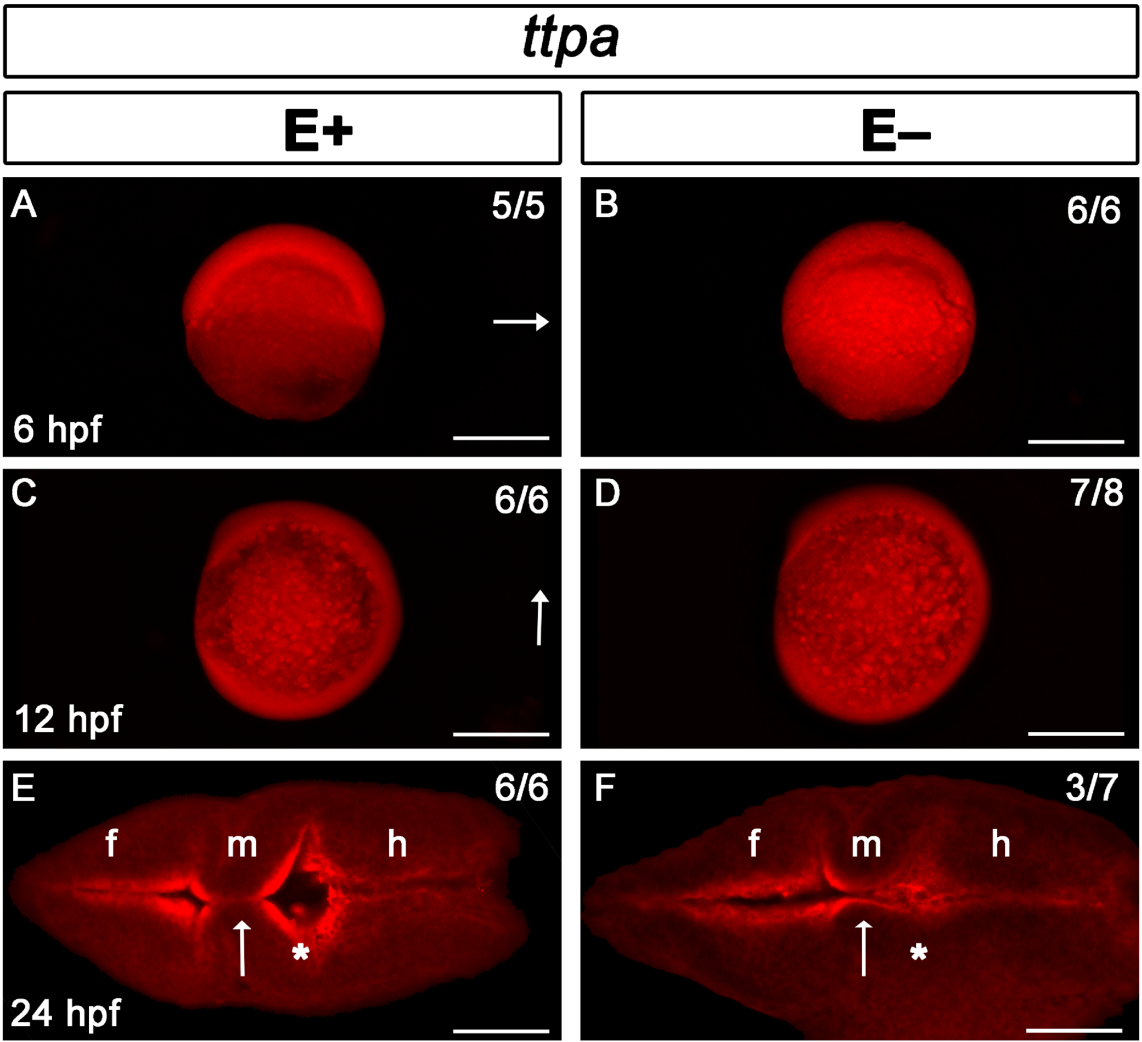
*Ttpa* signal localized throughout early embryo and brain ventricle borders regardless of VitE status. *Ttpa* expression in E+ and E− embryos is indicated with red fluorescence; dorsal direction is indicated by arrow (**A**–**D**). At 6 hpf (dorsal shield stage, **A**, **B**), *Ttpa* expression was present throughout the animal poles [E+ embryos, *n* = 5/5 (n = number of animals with the observed defect/total number of animals observed)], E− embryos, *n* = 6/6). At 12 hpf (90% epiboly, **C**, **D**), *ttpa* expression was present both in the embryo and in the yolk syncytial layer (E+ embryos, *n* = 6/6; E− embryos, *n* = 7/8 ), arrow indicates anterior region of the embryo. At 24 hpf (**E**, **F**), *Ttpa* expression was localized in the brain ventricle borders and within cells of the fore (f), mid- (m), and hindbrain (h). Arrows indicate the midbrain-hindbrain boundary where diencephalic ventricle expansion was altered; *Represent inflation in E+ embryos (**E**, *n* = 6/6) or lack thereof in E− embryos (**F**, *n* = 3/7). Scale bar represents 500 μm (**A–D**) and 50 μm (**E**, **F**); representative embryos are shown. Figure panels were generated with the BZ- × 700 microscope, processed with BZ-X Analyzer Software with image adjustments equally applied across time points in Adobe Photoshop v21.2.1.

We reported previously *ttpa* is necessary for zebrafish embryogenesis and its expression is localized to the most dorsal and anterior regions of the head and tailbud^13^. Because *ttpa* knockouts were lethal by 24 hpf, we hypothesized that *ttpa* expression is critical in regions requiring VitE delivery. To test this hypothesis, we compared *ttpa* expression in E+ and E− embryos. Surprisingly, at 6 hpf *ttpa* was ubiquitously expressed throughout the gastrulating animal regardless of VitE status (Fig. 2A,B). By 12 hpf, both E+ and E− embryos show similar *ttpa* expression patterns in the both the animal and yolk syncytial layer (Fig. 2C,D). By 24 hpf, *ttpa* expression was detected in the developing ventricles of the brain (Fig. 2E,F). VitE status did not appear to impact *ttpa* expression or its localization. However, VitE deficiency was associated with impaired brain development indicated by deformed mhb (arrow) and an uninflated third ventricle (*). Thus, VitE status did not regulate *ttpa* signal but was required for normal brain regionalization and structure by pharyngula stage prim-5 at 24 hpf when pigment formation also begins and the first heartbeat can be detected in zebrafish embryo^12^.

### Nervous system development markers

**Figure 3:**
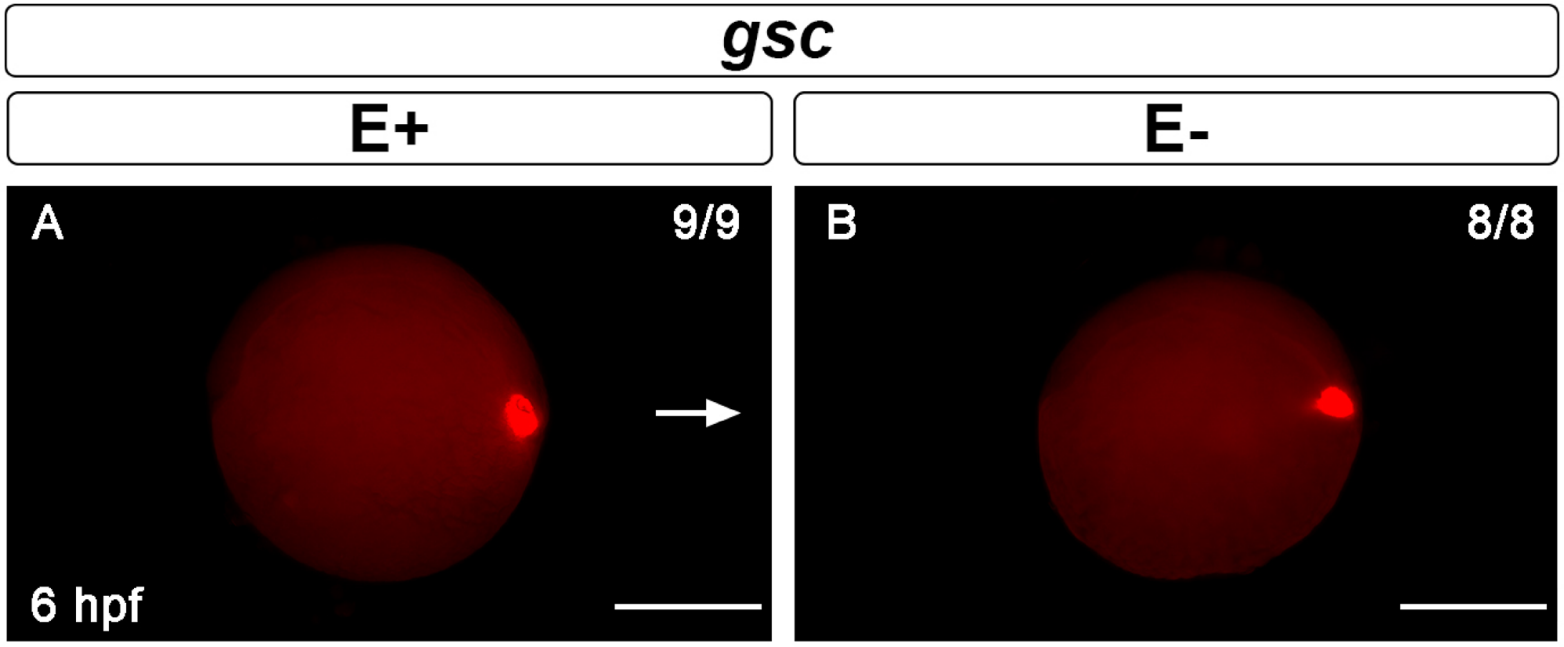
Gastrulation marker goosecoid (*gsc)* is not affected by VitE deficiency. *Gsc* expression at 6 hpf in the neuroectoderm of the dorsal embryonic shield, as indicated by red fluorescence in (**A**) E+ embryos (*n* = 9/9) and (**B**) E− embryos (*n* = 8/8). Arrow indicates dorsal region of the embryo. Scale bar represents 500 μm; representative embryos are shown. Figure panels were generated with the BZ- × 700 microscope, processed with BZ-X Analyzer Software with image adjustments equally applied across time points in Adobe Photoshop v21.2.1.

Early neurogenesis is defined by a specific set of transcriptional regulators that when present up- or down-regulate gene expression to cause proliferation and differentiation of neuronal tissue. Goosecoid (*gsc*), for example, is a nervous system patterning marker that indicates proper gastrulation and is expressed in the involuting neuroectoderm of zebrafish embryos at 6 hpf^24,48^. *Gsc* mRNA expressions at 6 hpf in E+ or E− embryos were not apparently different (Fig. 3) suggesting that VitE might not be critical for gastrulation at 6 hpf, a time point equivalent to mouse embryonic day 6 and human embryonic day 12^49^.

**Figure 4:**
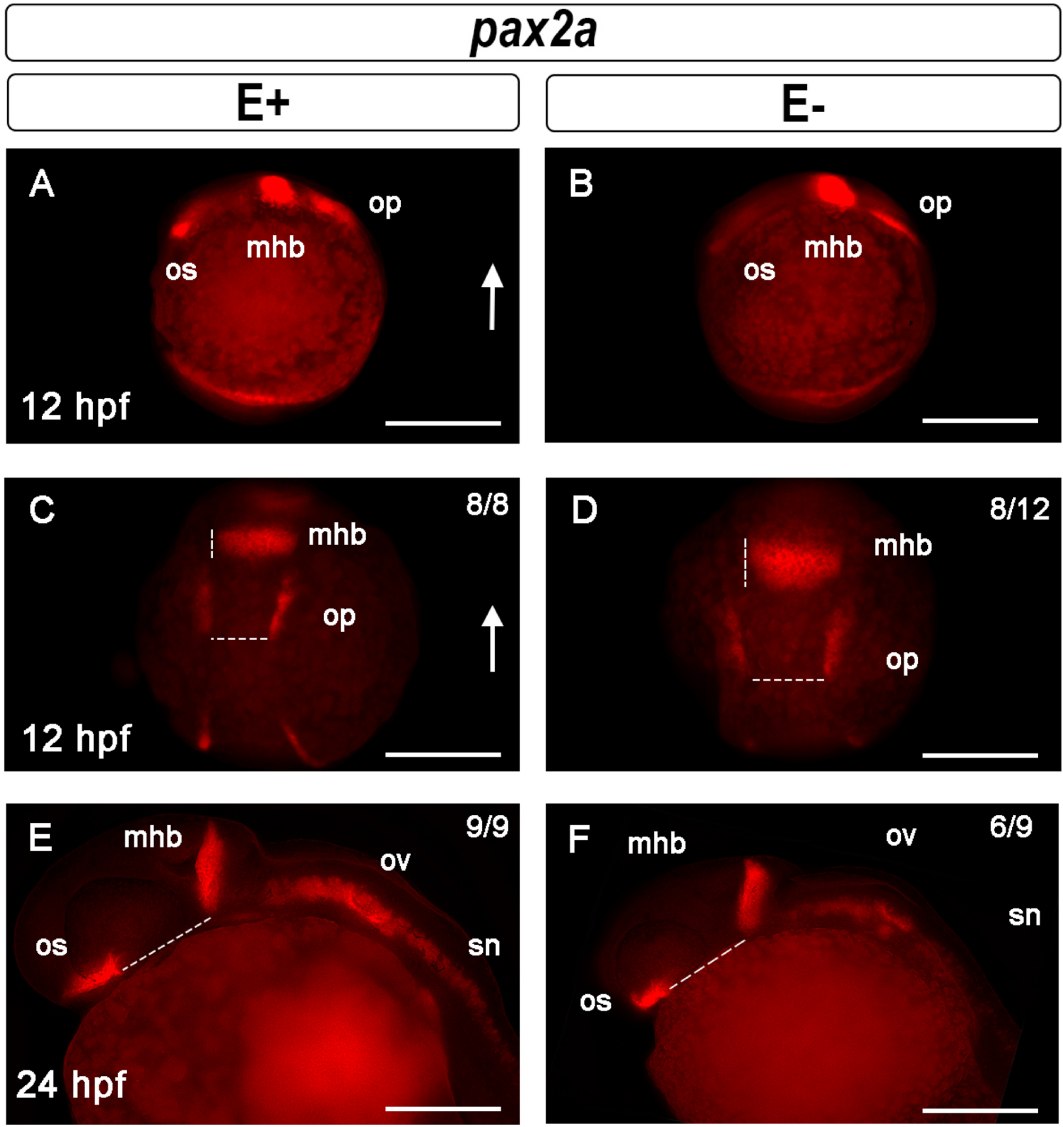
Midbrain-hindbrain boundary formation shown by *pax2a* expression is dysregulated by VitE deficiency. *Pax2a* expression in early optic stalk (os), midbrain-hindbrain boundary (mhb) and otic placode (op) in 12 hpf embryos, lateral views (**A**, **B**); arrow indicates dorsal region. At 12 hpf, E+ embryos (**C**) had defined mhb and op borders (*n* = 8/8), while E− embryos (**D**) had diffuse mhb and op borders (*n* = 8/12 assessed). Shown are representative E+ embryo with mhb 25 μm wide and op 49 μm apart, while representative E− embryo measurements were mhb 42 μm wide and op 63 μm apart. At 24 hpf, in E+ (**E**) and E− embryos (**F**) *pax2a* was expressed in the os, mhb, otic vesicles (ov) and spinal cord neurons (sn). Distance between os and mhb, a measure of first brain ventricle inflation, were greater in a representative E+ (91 μm) relative to an E− (80 μm) embryo. E+ embryo (**E**) spinal cord neurons at the same fluorescence exposure had significantly increased *pax2a* signal (*n* = 9/9), as compared with E− embryos (*n* = 6/9). Scale bar represents 100 μm; representative embryos are shown. Figure panels were generated with the BZ- × 700 microscope, processed with BZ-X Analyzer Software with image adjustments equally applied across time points in Adobe Photoshop v21.2.1.

Because early embryonic axis patterning was not impacted by inadequate VitE at 6 hpf, subsequent times (12 and 24 hpf) were investigated. *Pax2a* was used to evaluate the midbrain-hindbrain boundary (mhb) formation. At 12 hpf, E+ embryos have clearly developed mhb and otic placodes (op) (Fig. 4A,C). By contrast, at 12 hpf in E− embryos, the *pax2a* signal was ill-defined and diffuse, suggesting impaired formation of the mhb (Fig. 4C,D). Specifically, the width of the mhb in E− is 1.5 times greater than the mhb in E+ embryos. Moreover, the op margins in the E− were further apart than in the E+ embryos. These abnormalities may signal developmental delays, defects or both. By 24 hpf, *pax2a* is normally found in the anterior mhb, hind brain and spinal cord neurons^50^. In both E+ and E− embryos *pax2a* expression was prominent in the optic stalk (os) region of the forebrain (Fig. 4E,F). E− embryos had irregular *pax2a* expression characterized by a shortened distance between os and mhb. The *pax2a* expression pattern in the otic vesicle did not differ between E+ and E− embryos. By contrast, *pax2a* expression in spinal cord neurons (sn) was more abundant with structures more clearly defined in the E+ compared with the E− embryos (Fig. 4E,F). Overall, *pax2a* expression was most severely impacted in the mhb of E− 12 hpf embryos and in the spinal cord neurons of the E− 24 hpf embryos.

**Figure 5:**
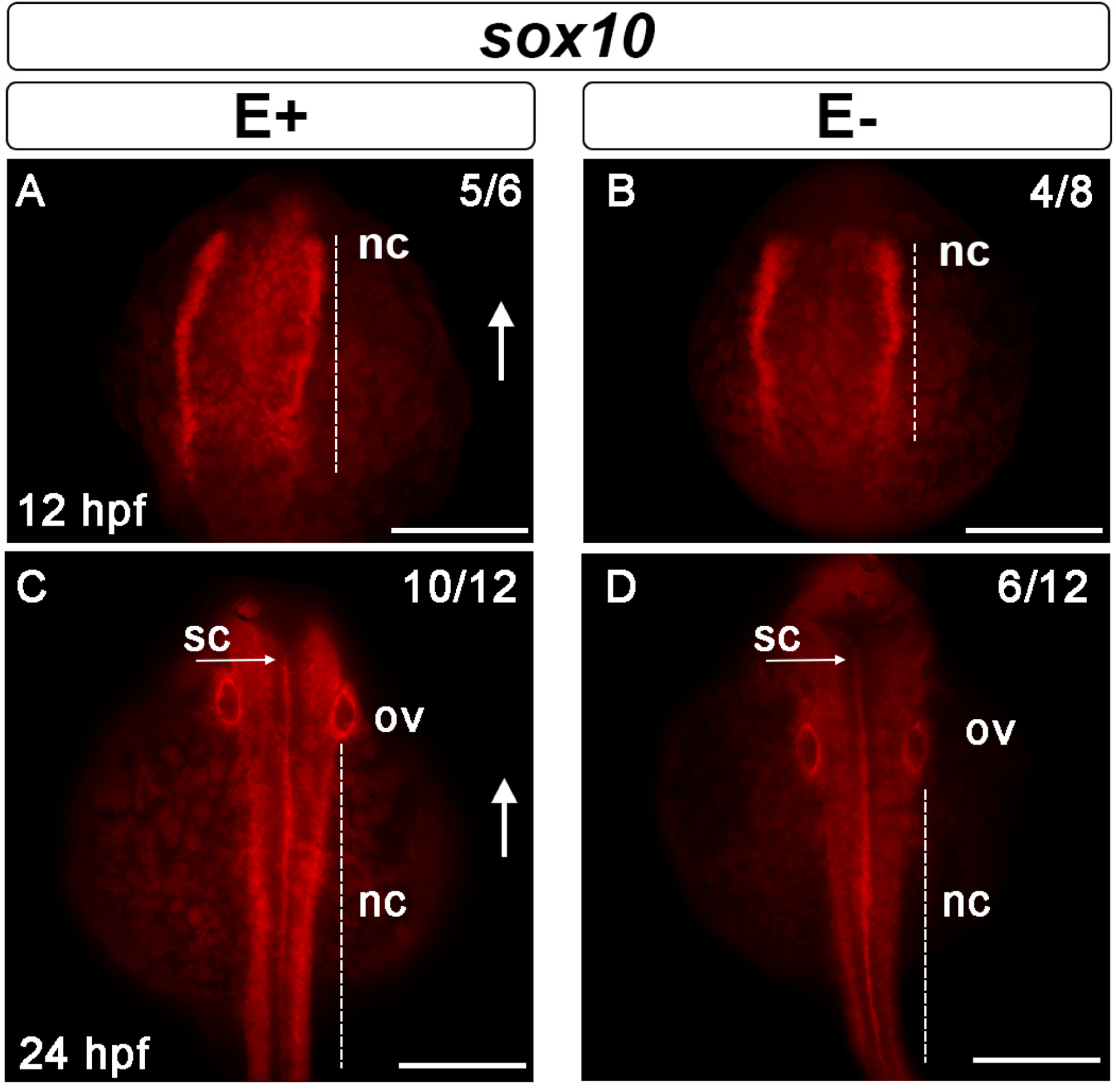
Neural crest cell migration impaired during development by VitE deficiency. S*ox10* was expressed in the cells of the neural border at 12 hpf (**A**, **B**); arrows indicate dorsal region of the embryo. The extent of the distribution of neural crest cells (nc) expressing *sox10* was greater (*n* = 4/8) in E+ embryos (**A**) relative to E− embryos (*n* = 5/6) (**B**). Measurements in representative embryos were E+ 174 μm relative to E− 145 μm. NC migrate ventrally away from the spinal cord (sc) to differentiate into cranial, cardiac, enteric and sensory neurons and glia. At 24 hpf, *sox10* expression around the otic vesicle (ov) was clearly defined in E+ embryos (**C**) and less well-defined in E− embryos (**D**). In E+ embryos (**C**), the migratory NC that flank the developing sc show increased *sox10* expression (*n* = 10/12), while E− embryos (**D**) had fewer migratory NC with *sox10* expression (*n* = 6/12). Nonetheless, similar spinal cord (s) *sox10* expression was observed in E+ and E− embryos. Scale bar represents 100 μm; representative embryos are shown. Figure panels were generated with the BZ- × 700 microscope, processed with BZ-X Analyzer Software with image adjustments equally applied across time points in Adobe Photoshop v21.2.1.

Sox10, another key transcription factor that plays a role in cell fate specification^51^, was evaluated at 12- and 24 hpf in E+ and E− embryos. The E+ embryos exhibited *sox10* expression pattern similar to the established patterns defined during zebrafish development^50^. E− relative to E+ embryos, however, demonstrate a restricted distribution of sox10 cell lineages in NC (Fig. 5A,B). By 24 hpf, *sox10* was abundantly expressed in E+ animals, in migratory NC, ov and down the spinal cord (sc) (Fig. 5C). Evidence of NC migration was observed by the increased *sox10* expression in the trunk of the animal into the dorsal root ganglia and early enteric neurons (not shown). E− embryos have reduced developmental progress relative to E+ based on the visibly different expression of sox10-expressing cells along the growing trunk with diffuse ov margins and reduced expression in NC. These findings were similar to the abnormal sn expression of *pax2a* in 24 hpf E− embryos, discussed above.

**Figure 6:**
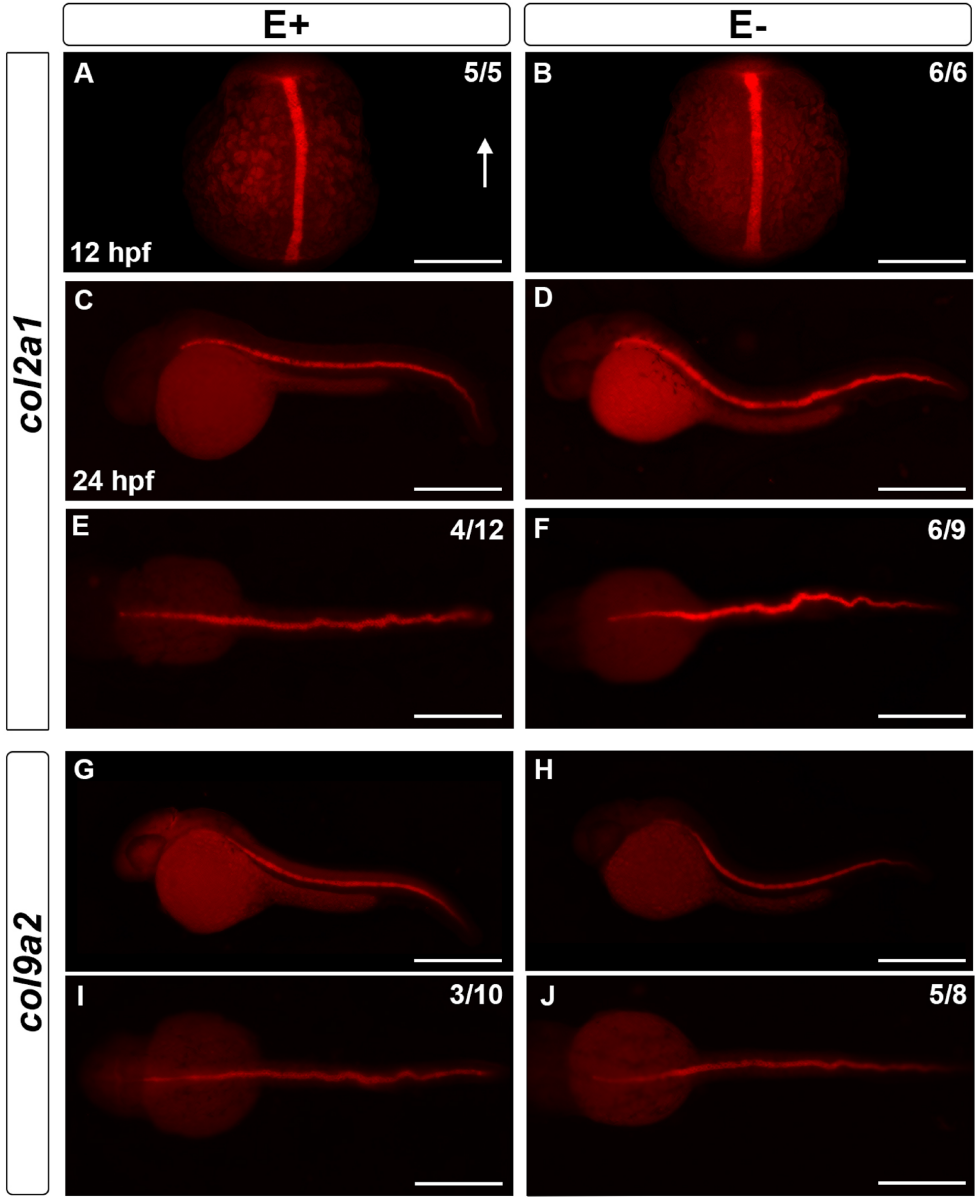
Notochord collagen markers *col2a1a* and *col9a2* affected by dietary treatment. At 12 hpf, *col2a1a* expression was localized in the developing notochord of E+ (**A**, *n* = 5/5) and E− (**B**, *n* = 6/6) embryos. Arrow indicates dorsal region of the embryo. At 24 hpf, the notochord sheath in E+ and E− embryos showed *col2a1a* (**C, D**) and *col9a2* (**G, H**) expression with a wavy notochord phenotype observed in both groups. The wavy notochord was more apparent when viewing dorsally (**E, F, I, J**) relative to lateral view (**C, D**, **G, H**). At 24 hpf, ~ 25% of E+ embryos (**E**, *n* = 4/12) showed a wavy *col2a1a* expression, while ~ 66% of E− embryos (**F**, *n* = 6/9) were obviously wavy; similarly a wavy *col9a2* expression was observed in 33% of E+ embryos (**I**, *n* = 3/10), while ~ 60% of E− embryos had a wavy expression (**J**, *n* = 5/8). Scale bar represents 500 μm (**C–J**) and 100 μm (**A, B**); representative embryos are shown. Figure panels were generated with the BZ- × 700 microscope, processed with BZ-X Analyzer Software with image adjustments equally applied across time points in Adobe Photoshop. This figure was created with Adobe Photoshop v21.2.1.

To determine the extent to which VitE status was associated with downstream neurogenesis targets, we used markers to localize notochord formation. The notochord is a conserved structural element and signal source point for neural tube and eventual spinal cord development^52,53^, where collagen synthesis, driven by *col2a1a* and *col9a2* expression, is critical. In the 12 hpf embryos, notochord formation did not vary by VitE status (Fig. 6A,B). By 24 hpf, however, both E+ and E− embryos showed notochord abnormalities with severe notochord bending more prevalent in E− embryos (Fig. 6C–J). The wavy notochord phenotype was present in some embryos from parents fed a standard lab diet (not shown) and may be an artifact of the fixative process. Similarly, we note that the notochord was more severely curved along the yolk axis in E− embryos and is not likely an artifact of fixation.

### Quantitation of gene expression

**Figure 7:**
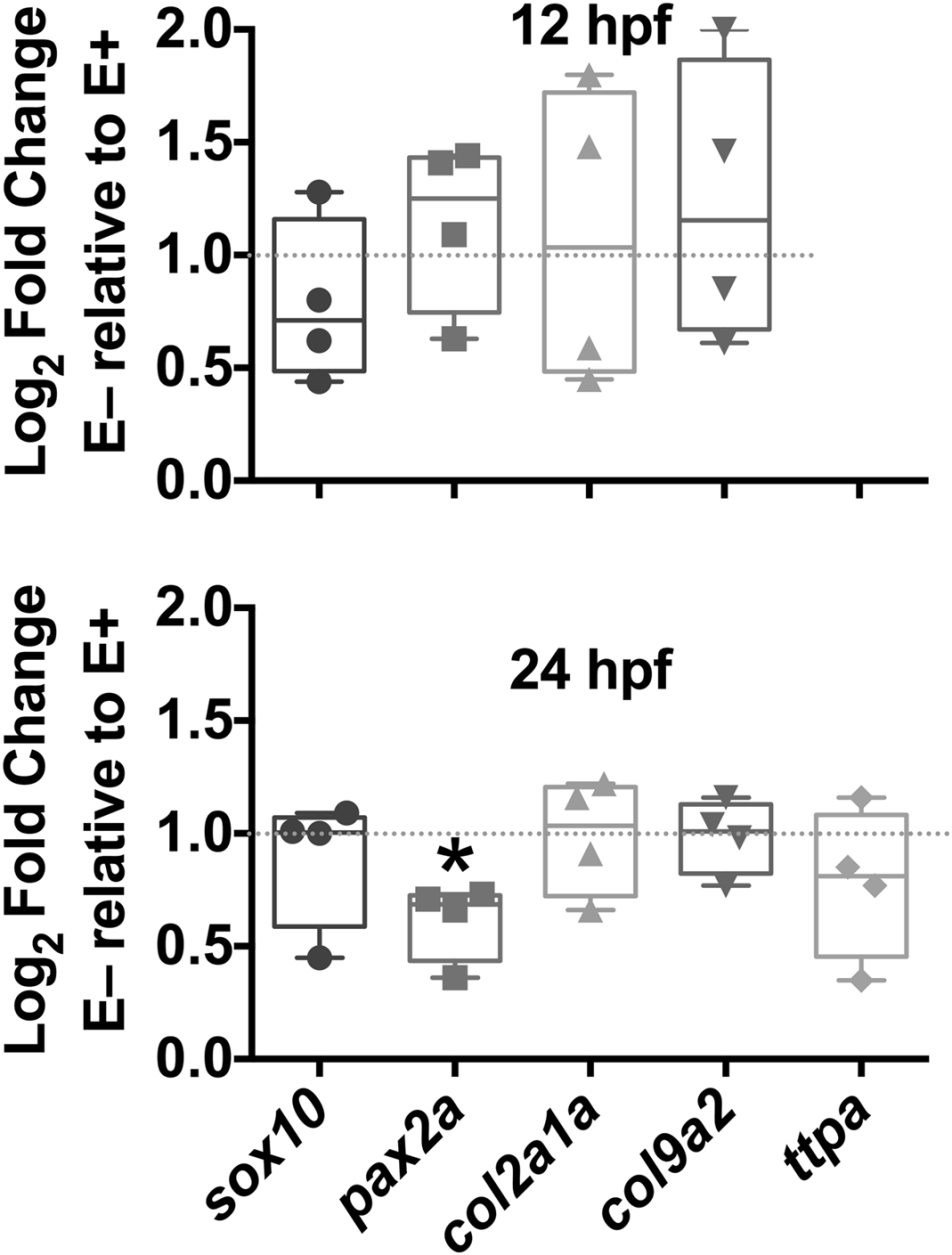
Relative gene expression of neurogenesis markers. Log_2_ fold change of genes measured in E− relative to E+ by RT-qPCR. *Ttpa* expression was not measured at 12 hpf. Data are as the median fold change, with the box from the 25th to the 75th percentile, the whiskers show the minimum and maximum value.*Indicates a significant difference in gene expression between E− and E+ Ct values.

To determine the extent to which VitE status altered the relative abundance of the same gene targets which had been localized, qPCR was performed. We hypothesized that transcriptional regulation would be altered and would be reflected in the patterning disruptions. Using relative fold change ratios (2^-ΔΔCt^), gene expressions at 12- or 24 hpf were evaluated in E− compared with E+ embryos (Fig. 7). Gene expressions of *sox10*, *pax2a*, *col2a1a* and *col9a2* were not significantly different at 12 hpf. At 24 hpf, only pax2a gene expression was lower (*P* < 0.05) in E− compared with E+ embryos. Thus, *Pax2a* expression was the only gene changed of those tested, which suggests that the mhb requires VitE for normal development.

### Histological analysis

**Figure 8:**
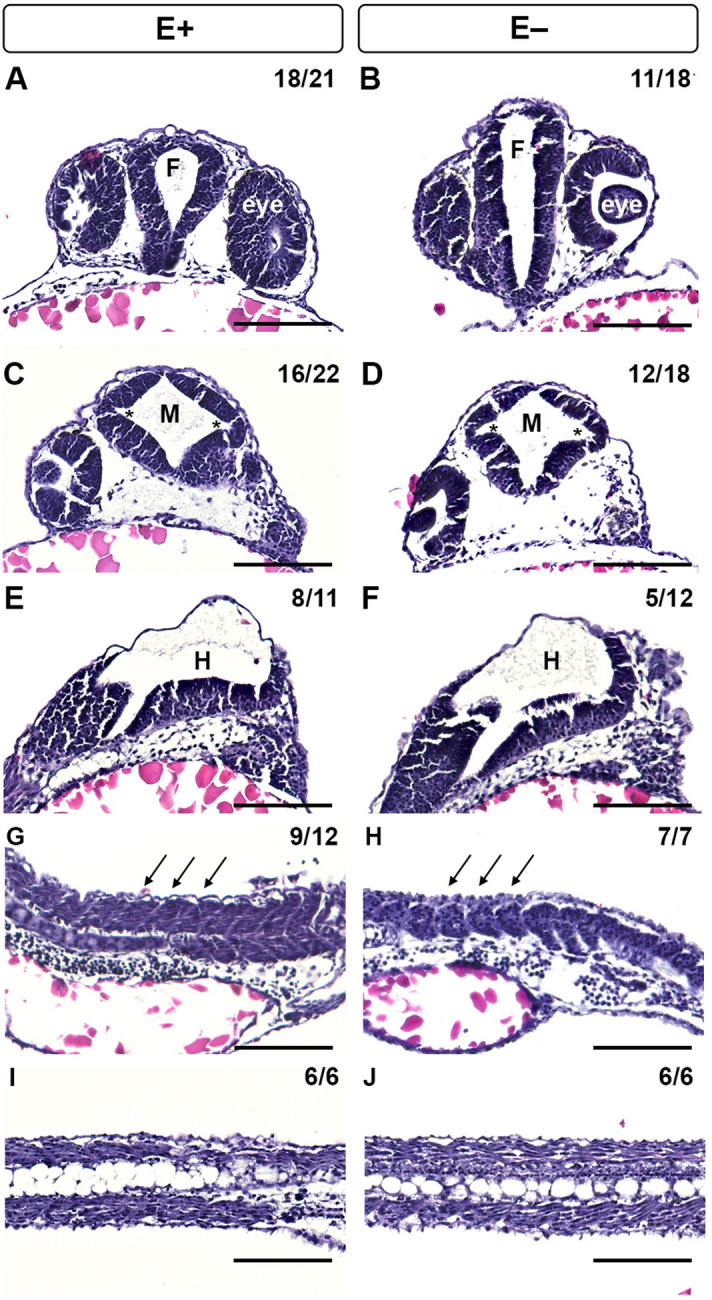
Histological analysis of 24 hpf zebrafish embryos with morphological defects associated with VitE status. Hematoxylin and eosin staining of E+ and E− embryos at 24 hpf was used to evaluate morphological defects, including fore- (F) and mid- (M), and hind- (H) brain ventricle inflation, somite formation and notochord vacuolation. Transverse section of E+ embryos had tear-drop shaped-F with eyes to each side (**A**), whereas E− embryos had a neural lumen, but improperly inflated-F (**B**). Serial transverse sections of E+ embryos showed normal M ventricle inflation with distinct hinge-points, indicated by *(**C**). E− embryos (**D**) had reduced M ventricle inflation with similar hinge points. E+ and E− embryos had similar H inflation leading to the neural tube in the trunk (**E, F**). Sagittal sections of the trunk indicate defined somite epithelial boundaries in E+ embryos (**G**) and indistinct epithelial boundaries in E− somitic cells (**H**). Notochord vacuolation appeared normal in both E+ (**I**) and E− (**J**) embryos. Scale bar represents 50 μm; representative embryos are shown. Figure panels were generated with the BZ- × 700 microscope, processed with BZ-X Analyzer Software with image adjustments equally applied across time points in Adobe Photoshop, v21.2.1.

Sectioned E+ and E− embryos at 24 hpf were hematoxylin and eosin stained to assess the structural effects downstream of the dysregulated gene express patterns (Fig. 8). E+ embryo (Fig. 8A) forebrains opened into a teardrop shape with eyes to each side, while E− embryos (Fig. 8B) exhibited an elongated ventricle with an improperly inflated lumen. In E+ embryos (Fig. 8C), the diencephalic ventricle at the midbrain opened into a diamond shape with distinct hinge points, while in E− embryos (Fig. 8D) the ventricles had somewhat distorted diamond shaped openings. The extent of hindbrain inflation was similar in E+ and E− embryos; however, roughly 72% were developed in the E+ (Fig. 8E) while only 41% were developed in the E− embryos (Fig. 8F). Somitic cells in E+ embryos (Fig. 8G) formed distinct, V-shaped epithelial boundaries, while in E− embryos somitic cells were loosely packed and formed U-shapes (Fig. 8H). The vacuolated notochord cells appeared normal regardless of VitE status (Fig. 8I,J).

## Discussion

Regardless of VitE status, *ttpa* is expressed throughout the developing zebrafish embryo at gastrulation as the neuroectoderm involutes forming the embryonic shield. Subsequently, *ttpa* was localized in the rostral head region of the zebrafish embryo at 24 hpf (Fig. 2), as we previously reported^13^. Specifically, *ttpa* was expressed at 24 hpf in the midbrain-hindbrain region and ventricle borders suggesting the critical need for the α-TTP in these regions of the early central nervous system (CNS). Because the known hepatic α-TTP role is to deliver VitE from recycling endosomes to the cellular membrane for local cellular redistribution^54^, our findings suggest that α-TTP in these brain regions is necessary to deliver VitE to newly formed neurons and/or differentiating surrounding neuronal cells. In adults, most neurons do not express *ttpa*, but Purkinje cells and associated Bergmann glia cells do^17^. Purkinje neurons and Bergmann glia cells are found in the cerebellum, a brain region critical for motor control and derived from the primordial hindbrain^55^. Shown here, *ttpa* was expressed at 24 hpf in the zebrafish embryo mhb. Our data suggest *ttpa* expression in the developing 24 hpf zebrafish embryo may be found in anatomically similar regions to where Purkinje progenitor neurons are found in rodent brain development^56^. We also showed that the lack of VitE did not change the abundance of *ttpa* expression as measured by qPCR, but its lack disrupted brain ventricle inflation and overall brain structure.

Little is known about VitE trafficking in the brain. Our findings support the hypothesis that VitE is necessary in the midbrain-hindbrain boundary to support formation of the ventricular shape, size and constriction, which may be a result of two different VitE functions. First, VitE is a potent lipophilic antioxidant that is a necessary antioxidant to protect the polyunsaturated fatty acid-rich membranes of nervous tissue^57^. Second, VitE enhances membrane fluidity and repair^58^. The developing brain undergoes significant membrane expansion and growth requiring a degree of neuronal plasticity facilitated by membrane composition^59^, and our work supports the idea that VitE serves critical roles during this process. The histological analysis further supports that VitE is necessary during somitic cell formation, which is highly dependent on convergence and extension of the lateral mesoderm^60^. E− embryos display somitic defects consistent with impaired convergence and extension, which may further impact neural tube formation and closure by 24 hpf. Collagen sheath markers *col2a1a* and *col9a2* (Fig. 6D,H) also demonstrate that E− embryos had severely bent axes. Similarly, the *ttpa* morpholino-knockout embryos displayed a shrunken body axis^61^, also indicating dysregulated convergent extension. Patterns of genes associated with convergent extension will be investigated in the future based on the mounting evidence regarding NTDs, body axis development and the VitE-deficient phenotype shown throughout this study.

Histological analysis also validates morphological deformations observed by whole mount in situ hybridization. Both techniques demonstrated that the forebrains in E− embryos had ill-defined boundaries and were overly inflated (Figs. 2F, 8B). Similarly, the E− midbrain ventricles had abnormal morphology with neuroepithelial bending at the hingepoints (Figs. 2F, 8D). Somitogenesis plays a critical role in determining NC cell migration^62^. Errors in somite formation observed in E− embryos (Fig. 8H) may explain the reduced signal of *sox10* in NC cells migrating ventrally along the zebrafish midline and primordial spinal cord (Fig. 5D).

Because *ttpa* was expressed from 6- to 24 hpf (Fig. 2), VitE must be essential in the same developmental period, especially in the structures that express *ttpa*. We aimed to determine at what period embryonic development goes awry. To investigate gastrulation, which occurs at nearly 50% epiboly with the formation of the embryonic shield^24,63^, we evaluated *gsc* expression^63^, but found that *gsc* was not affected by VitE status. Interestingly, Niemann-Pick C Disease-1 depleted embryos have impaired VitE trafficking and also have normal *gsc* expression at the same early timepoint^64^. Although modulation of lipid metabolism did not affect embryonic *gsc* expression^65^; a ventralizing effect at the shield stage was observed with *gpx4b* knockdown, which increases oxidative damage by impairing lipid hydroperoxide detoxification^66^. E− embryos undergo normal gastrulation, which suggests that the oxidative damage due to VitE deficiency is insufficient to cause very early stage abnormalities. Thus, VitE appears to be needed at a subsequent time point, such as during the formation of the neural keel, rod folding and neural tube cavitation, which occur between 12- and 24 hpf.

Genetic markers associated with NC proliferation, migration and the formation of the mhb were also studied. *Sox10* expression is found in zebrafish embryo NC and their progenitor cells preceding formation of NC-derived sensory neurons and dorsal root ganglia^33^. Mislocated *sox10* expression patterns were observed at 12- and 24 hpf in E− embryos (Fig. 5). These patterns have been seen in zebrafish embryos with folate deficiency-induced neuropathy, which was associated with irregular *sox10* expression at similar developmental periods^43^. *Pax2a* defines the mhb, or isthmic organizer, a constricted portion of the still open neural tube that coordinates patterning in both the midbrain and cerebellum^28^. Localization of the *pax2a* gene in the E− embryo at two developmental timepoints shows that the mhb is ill-formed at 12 hpf (Fig. 4D) and subsequently structures critical for the peripheral nervous system, including spinal cord neurons, are less abundant (Fig. 4F). These data suggest that oxidative damage may be impairing neurodevelopment. VitE deficiency causes lipid peroxidation resulting in whole embryo metabolic disruption including increased phospholipid turnover and choline utilization with subsequent methyl donor depletion^3,67^. Folic acid deficiency similarly depletes methyl donors producing similar *pax2a* expression pattern errors to those described herein in the E− embryos^43^. We suggest that VitE deficiency may act in a similar manner by depleting methyl donors and thus disrupt brain development because we found *pax2a* gene expression was also mislocated and quantitatively reduced (Fig. 7B). Further study is needed to explore the mechanisms involved.

This study shows that VitE deficiency causes severe developmental impairment at early embryonic stages. VitE does not regulate *ttpa* expression patterns but does alter the tissue structures where it is found (Fig. 2D,F). Major pattern disruptions at 12 and 24 hpf, as indicated by *pax2a* and *sox10*, may indicate VitE-dependent regions of the developing nervous system. In addition to disrupted brain regionalization and NC migration, E− embryos experience severe morphologic abnormalities indicated by bent axes as indicated by *col2a1a* and *col9a2*. Additionally, histological evidence agrees with the dysregulated gene expression patterns and brain morphology in E− embryos, indicating impaired brain ventricle inflation and somite formation. These experiments represent a major step in determining the molecular basis of VitE in the developing vertebrate nervous system.

## Methods

### Zebrafish husbandry

All experimental protocols and methods were carried out in accordance with the animal use and care protocol (# 5,068) approved by the Institutional Animal Care and Use Committee at Oregon State University. Tropical 5D strain zebrafish were reared in Sinnhuber Aquatic Research Laboratory at Oregon State University under standard laboratory conditions of 28 °C on a 14 h light/10 h dark photoperiod according to standard zebrafish breeding protocols^12^. At 55 days post-fertilization (dpf), adult zebrafish were randomly allocated to two experimental diets, vitamin E deficient (E−) or sufficient (E+), as described^61,68,69^.

Vitamin E was extracted from the diets prior to feeding and assayed by HPLC–UV prior to feeding, as described^70^. In the E+ diet, α- and γ-tocopherols were 229.8 ± 8.1 and 16.5 ± 0.5 mg/kg ± SEM (n = 5 measurements), respectively. In the E− diet, α- and γ-tocopherols were 6.3 ± 0.2 and 2.4 ± 0 mg/kg, respectively. E− and E+ embryos were obtained by group spawning of adult fish fed either E− or E+ diets for a minimum of 80 days up to 9 months. Embryos were collected, staged and incubated until use in standard embryo media (15 mM NaCl, 0.5 mM KCl, 1 mM MgSO_4_, 0.15 mM KH_2_PO_4_, 0.05 mM Na_2_HPO_4_, 1 mM CaCl_2_, NaHCO_3_ in fish system water).

### Morphological and histological assessment

At each time point (12-, 24-, 48 hpf), embryos are assessed for morbidity and mortality outcomes, as described^71,72^; at 12 hpf, embryos are assessed for viability and developmental progression if development exceeds 3 h the expected stage. 24- and 48 hpf embryos are assessed similarly with more attention to embryonic morphological outcomes, including brain, eye, somite and notochord formation as well as early pericardial and yolk sac edema onset. 24 hpf embryos from both diet groups were euthanized with tricaine (MS222, ethyl 3-aminobenzoate methanesufonate salt, Sigma Aldrich) in accordance to animal care and use guidelines, fixed overnight in 4% paraformaldehyde (PFA) solution, embedded in paraffin blocks, serially sectioned and stained with Hematoxylin and Eosin for histological assessment (Oregon Veterinary Diagnostic Lab, Corvallis, OR). Live imaging of embryos was conducted using a Keyence BZ- × 700 All-in-one microscope with a 2 × or 10 × objective. Embryos were mounted in 3% methylcellulose at room temperature in 35 mm glass bottom MatTek dishes and covered in embryo media and tricaine to anesthetize embryos. Image adjustments, including cropping, brightness and contrast were performed using Adobe Photoshop.

### Whole mount in situ hybridization

The whole mount in situ hybridization protocol with minor modifications was used as described^50,73^. Briefly, RNA was extracted from pooled E+ or E− embryo lysates. cDNA was synthesized using the High Capacity SuperScript kit (Applied Biosystems). Primers were designed for a 1 kb portion of the 3′ UTR region of gene targets of interest and contained an RNA polymerase promoter sequence, either T3 or T7, located at the 5′ end of the reverse primer to make an antisense RNA probe (Supplementary Table 1). DNA templates were prepared using PCR amplification followed by synthesis of antisense RNA probes utilizing an in vitro RNA transcription reaction kit with DIG-UTP labeled nucleotides. PCR product and RNA probe quantity and quality were checked at each step by electrophoresis on 1.2% agarose gels and spectrophotometrically with a BioTek3 plate reader. A hybridization buffer was prepared with 50% formamide, 5X sodium saline citrate (SSC), 50 μg/mL heparin, 500 μg/mL tRNA, 0.1% Tween, and 9 mM citric acid in RNAs E−free water.

Embryos used for in situ hybridization techniques were euthanized by tricaine prior to fixation, in accordance to animal care and use guidelines. Embryos at the desired time points were dechorionated by hand using sharp forceps, then fixed overnight in 4% PFA. Fixed embryos were washed 3 times with sterile phosphate buffered saline (PBS) and stored in methanol at − 20 °C. For analysis, embryos were rehydrated in PBS, permeabilized with proteinase K (10 μg/mL), fixed with 4% PFA and hybridized with antisense probe in the hybridization buffer with 10% dextran sulfate. The following day, embryos were washed PBS with 0.1% Tween and incubated with 1:5,000 anti-DIG-Alkaline Phosphatase Label (Roche) in blocking buffer [2% sheep serum (vol/vol) and bovine serum albumin (2 mg/mL) in PBS with 0.1% Tween] on a gentle rocker at 4 °C overnight. Embryos were then washed with Fast Red Buffer [filter (0.2 μm) sterilized, Tris HCl (36 mM) and NaCl (2 M) in sterile water at pH 8.2]. Finally, color development was observed with Fast Red (Sigma Aldrich) to detect alkaline phosphatase-digoxigenin tagged probes and generate an insoluble red precipitate and rhodamine fluorophore. Fixed embryo imaging was conducted using a Keyence BZ- × 700 All-in-one microscope with a 10 × or 20 × objective. Embryos were mounted in 3% methylcellulose at room temperature in 35 mm glass bottom MatTek dishes. All images from the same time point and probe were taken with the same exposure in the same optical plane and processed with the BZ-x Analyzer Software (Keyence). Additional image adjustments, including cropping, brightness and contrast applied were performed using Adobe Photoshop.

### Relative reverse transcription PCR

RNA was extracted from embryos staged developmentally at 12 and 24 hpf, pooled (n = 10 embryos/sample) and homogenized; 4 sample pools per VitE status. RNA integrity (9.02 ± 0.15, n = 16) was assessed using the 2,100 BioAnalyzer Instrument (Agilent) at the Center for Genome Research and Biocomputing at Oregon State University. Reverse transcription to synthesize cDNA was performed with the High Capacity Superscript Kit (Applied Biosystems). Primers were designed for 100–200 bp amplicons in the 3′ UTR region of the target genes (Supplementary Table 1). PCR was performed with final concentrations 1X Sso Advanced Universal SYBR Green Supermix (Bio-Rad), 500 nM forward and reverse primers and ~ 20 ng template.

### Statistical analyses

All statistical analyses were evaluated using Prism (GraphPad Software Inc., CA). Viability outcomes assessed linear regression with comparison of slopes. Gene expression data were normalized using 18S ribosomal subunit expression and analyzed with the 2^-ΔΔCt^ method, as described^74^. Differences between groups, evaluated by T-test were determined to be statistically significant if *P* < 0.05.

## Supplementary information

Supplementary Information.

## Abbreviations

α-TTP: α-Tocopherol transfer protein
NTD: Neural tube defects
VitE: Vitamin E
E+: VitE sufficient
E−: VitE deficient
Hpf: Hours post-fertilization
