# Supplementary material for: Vitamin E is necessary for zebrafish nervous system development
Source: Sci Rep. 2020 Sep 21;10:15028. doi: 10.1038/s41598-020-71760-x (PMC7506018; doi:10.1038/s41598-020-71760-x)
Supplement: Supplementary file 1 — Supplementary Information. [file 41598_2020_71760_MOESM1_ESM.pdf]

**Vitamin E is necessary for zebrafish nervous system development**

Brian Head[1,2], Jane La Du[3], Robyn L. Tanguay[3], Chrissa Kioussi[4], Maret G. Traber[1,5]*

**Supplementary Table 1. Primer features for WISH and RT-qPCR.**

Primers for WISH were designed to be >700bp for enhanced specificity to target mRNA. UPPERCASE letters denote the T7 or T3 polymerase promoter site for *in vitro* synthesis reactions of antisense RNA strand. PCR primers are designed for regions <200bp and were tested for specificity by melt-curve analysis.

| Gene name | Forward primer | Reverse primer | Amplicon size (bp) |
|---|---|---|---|
| *ttpa* | | | |
| WISH | tgcgtggcatccacttgata | CATTAACCCTCACTAAAGGGAAaagaatcggagcagaaggca | 886 |
| PCR | cctcatccgtcattggactt | tttggggttccatttaccaa | |
| *gsc* | | | |
| WISH | tcttttcgcttgtgatgccc | TAATACGACTCACTATAGGGtcagctgtcagaatccacgt | 737 |
| *sox10* | | | |
| WISH | acctaccgaagtcacctgtg | TAATACGACTCACTATAGGGgtgactctgacctgtagcgt | 754 |
| PCR | tgaacgagacggataagcgg | tgcaggctcttgtaatgcga | |
| *pax2a* | | | |
| WISH | acatgatctgcacctgacca | TAATACGACTCACTATAGGGattggaagcgcttgacacag | 748 |
| PCR | gtgacaggtcgagagatggc | acttaataacgcggggttgct | |
| *col2a1a* | | | |
| WISH | cgttacatgcgtgcagatga | TAATACGACTCACTATAGGGtgatctgctcctccgatgtc | 716 |
| PCR | gtgtgtgattcggggactgt | tttgcaccaagtgacccgat | |
| *col9a2* | | | |
| WISH | ttctggggtgttgggagatc | TAATACGACTCACTATAGGGggacccttgatcacctgtca | 838 |
| PCR | tctgcttggccttatcgcaa | cccgactctcccttttgacc | |
